# Mapping the Interactive Effects of ApoE Gene Polymorphism on Caudate Functional Connectivity in Mild Cognitive Impairment Associated With Parkinson’s Disease

**DOI:** 10.3389/fnins.2020.00857

**Published:** 2020-09-17

**Authors:** Song’an Shang, Yu-Chen Chen, Hongying Zhang, Weiqiang Dou, Long Qian, Xindao Yin, Jingtao Wu

**Affiliations:** ^1^Department of Radiology, Nanjing First Hospital, Nanjing Medical University, Nanjing, China; ^2^Department of Radiology, Clinical Medical College, Yangzhou University, Yangzhou, China; ^3^GE Healthcare, MR Research China, Beijing, China

**Keywords:** Parkinson’s disease, cognitive deficits, magnetic resonance imaging, genetic polymorphism, APOE

## Abstract

**Introduction:**

Cognitive impairment (CI) is a frequent non-motor symptom of Parkinson’s disease (PD). Caudate and Apolipoprotein E (ApoE) are biomarkers linked to CI in PD. There is little known about whether ApoE affects caudate in mild CI of PD (PD-MCI). We investigated the possible interactive effect of ApoE genotypes on caudate functional connectivity (FC) in PD-MCI.

**Methods:**

A total of 95 PD-MCI patients and 99 matched healthy controls underwent extensive neuropsychological assessment and magnetic resonance imaging. The two groups were separated into three subgroups according to their genotyping. Functional data were analyzed with FC analysis.

**Results:**

Decreased FC between the caudate and the bilateral inferior orbit frontal gyrus and bilateral middle occipital gyrus (MOG) was found between groups, along with poor performance in general, executive, episodic memory, language, and visual–spatial function. Decreased FC between the caudate and right MOG, right middle temporal gyrus, and right superior occipital gyrus was found as an interaction effect. The FC values of ε4 carriers with PD-MCI were much lower than the other carriers, and FC was positively correlated with the impairment of global and language function.

**Conclusion:**

These results support the idea that altered FC between the bilateral caudate and posterior cortical regions was interactively influenced by ApoE genotype and PD-MCI status, and the ε4 subtype associated with underlying pathology of global cognitive decline and semantic fluency impairment in an interactive manner. Gene-based imaging approaches might strengthen the credibility in imaging genetic associations, which might provide new powerful insights into the neural mechanisms underlying PD-MCI.

## Introduction

Cognitive impairment (CI) is a prevalent non-motor symptom of Parkinson’s disease (PD) and an important cause of disability in that condition; the severity of this symptom ranges from mild cognitive impairment (MCI) to dementia (Svenningsson et al., 2012). PD combined with MCI (PD-MCI) is identified as slight CI without functional decline during the progression of PD (Litvan et al., 2012). Compared to the absence of cognitive dysfunction, the early presence of PD-MCI markedly increases the incidence (found in up to 80% of cases) of PD dementia (PDD) at later stages of the disease (Hely et al., 2008; Litvan et al., 2011). Thus, the concept of PD-MCI is not only useful for understanding CI in PD but also critical for diagnosis and clinical interventions.

The hallmark pathology of PD consists of neurotransmitter system dysfunction (dopaminergic or cholinergic) and Lewy body deposition (cortical and subcortical) (Leverenz et al., 2009; Dickson, 2018). Neuroimaging studies have revealed that these neurochemical alterations induce impairment of neuronal processing in the basal ganglia (Haber, 2003; Montgomery, 2015), especially the neostriatum, which is the key hub of cortico-striato-thalamo-cortical loops (Brooks and Piccini, 2006). However, the cognitive symptoms of PD differ dramatically from the motor symptoms in the early stages. Manza et al. (2016) observed that cognitive deficits in PD-MCI were associated with strengthened functional connectivity between the caudate and anterior cingulate cortex, whereas a higher motor deficit rating was associated with weaker functional connectivity between the putamen and midbrain. Hence, the caudate nucleus plays a vital role in cognitive changes in PD, and compelling evidence suggests that this structure provides a pathological biomarker for the mechanisms underlying cognitive decline in PD-MCI.

Genetic polymorphisms associated with cognitive decline have been shown to provide critical insight into the biological mechanism of heterogeneity in PD-MCI. Despite some inconsistency, recent epidemiological genome-wide association studies have suggested that apolipoprotein E (ApoE) is a genetic vulnerability biomarker linked to cognitive decline in PD (Morley et al., 2012; Tropea et al., 2018; Sun R. et al., 2019), as opposed to a risk factor for the etiology of PD (Peplonska et al., 2013; Multhammer et al., 2014). Compared to MCI in Alzheimer’s disease (AD), PD-MCI frequently includes an increased severity of executive and visuospatial deficits and a decreased severity of memory impairment (Aarsland, 2016; Jellinger and Korczyn, 2018). Moreover, considering that ApoE is a well-recognized susceptibility gene for AD (Sun Y. et al., 2019), it is still far from clear whether the effects of this gene are different in PD patients.

rs-fMRI is a particularly appealing technique, since its images can be combined with genetic polymorphism information to characterize the influence of genetic factors on brain activities. A recent study conducted by Zhang et al. (2019) showed, by analyzing the amplitude of low-frequency fluctuation, that an alpha-synuclein (SNCA) gene polymorphism (rs894278) and PD status interactively affect the brain activity of PD patients and controls, but it did not focus on the cognitive alterations that make up PD-MCI. Rittman et al. (2016) found a link between regional microtubule-associated protein tau (MAPT) expression and selective vulnerability of functional brain networks to neurodegeneration of PD; unfortunately, that study did not examine ApoE gene polymorphism.

The underlying functional communication between different anatomical brain regions is reflected by functional connectivity (FC) analysis (van den Heuvel et al., 2009). There is little research on whether the ApoE gene affects caudate FC in a way that contributes to cognitive decline in PD-MCI. Thus, in the present study, the possible effect of different ApoE genotypes on caudate FC in PD-MCI was investigated, and we hypothesized that this effect interacts with the pathogenesis of PD.

## Materials and Methods

### Participants

From May 2017 to February 2019, we recruited 194 subjects (all right-handed Chinese individuals) through normal community health screening, newspaper advertisements, and hospital outpatient services; these subjects from the original sample comprised 95 (52 male and 43 female) patients diagnosed with PD-MCI and 99 (49 male and 50 female) healthy controls (HC) matched for age, sex, and years of education. This study was performed with approval from the local institutional review board of Clinical Medical College, Yangzhou University (2015KY-081), and written informed consent was obtained 24 h prior to the examinations.

The inclusion criteria (Amboni et al., 2015) for the PD-MCI group were as follows: (1) subjects met the PD-MCI diagnosis according to both United Kingdom Parkinson’s Disease Society Brain Bank Clinical Diagnostic Criteria (Hughes et al., 1992) and level I of Movement Disorders Society Task Force criteria (Litvan et al., 2012); (2) subjects were in an early clinical disease stage, Hoehn and Yahr (H&Y) stage ranging from I to II while in the “ON state” according to the initial assessment; (3) subjects fulfilled at least two of the criteria of bradykinesia, rigidity, and resting tremor, or they had asymmetric resting tremor or asymmetric bradykinesia; (4) subjects had been taking a stable and optimized daily dose of antiparkinsonian medications for at least 4 weeks prior to study entry. Additionally, all patients abstained from antiparkinsonian drugs for at least 12 h before the clinical assessments (including motor and cognitive scales) and MRI acquisition. Control subjects were required to have a Montreal Cognitive Assessment (MoCA) score of ≥26.

The following exclusion criteria were used for all participants: (1) family history of PD, secondary parkinsonism, or Parkinsonism syndrome; (2) any neurological disease such as AD, depression, or dysthymic disorder, or any psychiatric disease; (3) any disease related to the nervous system, including major head injury (with loss of consciousness or other complications), cerebrovascular disorders, epilepsy, brain tumors, diabetes, alcoholism, or neurological surgery; (4) use of psychotropic agents, anticholinergic drugs, or other treatments potentially interfering with cognition; (5) any contraindication for MR imaging, including claustrophobia, ferromagnetic foreign bodies, and electronic implants; (6) severe vision or hearing loss; and (7) excessive head motion found in data preprocessing. Participant demographic and clinical data are shown in Table 1. The inclusion and exclusion assessments were performed by two experienced neurologists (Yao Xu, with 31 years of experience, and Hengzhong Zhang, with 35 years of experience), who administered a structured interview to subjects and their informants.

**TABLE 1:** Demographic and clinical characteristics of the participants included in the study.

	PD-MCI (*n* = 95)			HC (*n* = 99)			Diagnosis	Gene	Interaction
	ε2 (*n* = 21)	ε3 (*n* = 46)	ε4 (*n* = 28)	ε2 (*n* = 31)	ε3 (*n* = 42)	ε4 (*n* = 26)	*P* (*F*) value	*P* (*F*) value	*P* (*F*) value
Age	66.29 ± 12.01	65.17 ± 10.80	63.83 ± 7.39	62.75 ± 10.82	62.82 ± 9.97	62.38 ± 10.27	0.21 (1.59)	0.87 (0.14)	0.73 (0.32)
Gender (M/F)	9/12	26/20	17/11	19/12	19/23	11/15	0.58 (0.31)	0.38 (0.96)	0.71 (0.35)
Education (years)	13.06 ± 3.61	12.78 ± 1.12	12.75 ± 3.63	13.00 ± 4.52	13.71 ± 3.86	12.48 ± 4.44	0.79 (0.072)	0.74 (0.30)	0.69 (0.38)
Disease course (years)	5.76 ± 4.81	5.24 ± 2.78	6.25 ± 2.17	–	–	–	–	–	–
UPDRS	45.41 ± 17.43	43.74 ± 11.57	48.42 ± 11.56	–	–	–	–		–
H-Y stage	1.79 ± 0.75	1.77 ± 0.59	1.79 ± 0.43	–	–	–	–		–
LEDD (mg/day)	427.84 ± 314.72	452.41 ± 310.55	437.28 ± 304.81	–	–	–	–		–
MoCA	22.94 ± 1.80	22.48 ± 1.91	21.25 ± 1.74	28.50 ± 1.12	28.39 ± 1.36	28.47 ± 1.40	0.0010(39.88)*	0.12 (2.19)	0.095 (2.38)
TMT-A	84.35 ± 34.41	90.95 ± 39.43	83.75 ± 56.71	71.83 ± 40.99	75.73 ± 33.73	82.62 ± 51.77	0.20 (1.65)	0.82 (0.19)	0.69 (0.37)
TMT-B	185.53 ± 53.06	182.91 ± 49.68	225.33 ± 43.43	168.08 ± 60.49	149.55 ± 66.28	155.29 ± 63.81	0.001(13.87)*	0.11 (2.20)	0.20 (2.20)
Similarity	15.82 ± 3.11	15.80 ± 4.26	13.17 ± 3.05	16.25 ± 3.92	17.53 ± 4.53	16.48 ± 4.04	0.020(5.51)*	0.092 (2.42)	0.42 (0.86)
HVLT-R (Total)	19.29 ± 5.54	19.61 ± 6.05	16.75 ± 5.46	21.25 ± 8.84	22.26 ± 7.91	20.05 ± 7.12	0.044(4.11)*	0.21 (1.59)	0.94 (0.066)
HVLT-R (Delayed)	7.12 ± 2.74	7.18 ± 2.93	6.75 ± 2.52	7.92 ± 2.56	7.44 ± 2.65	6.90 ± 3.04	0.44 (0.60)	0.61 (0.49)	0.89 (0.12)
HVLT-R (Recognition)	8.53 ± 2.23	8.61 ± 2.43	7.58 ± 1.98	8.42 ± 2.10	9.27 ± 2.18	8.62 ± 2.52	0.22 (1.54)	0.17 (1.82)	0.62 (0.48)
VFT (semantic)	13.71 ± 2.89	12.76 ± 2.28	11.83 ± 2.11	15.42 ± 2.81	15.53 ± 2.95	15.24 ± 2.74	0.001(28.26)*	0.33 (1.10)	0.47 (0.75)
VFT (phonemic)	7.47 ± 2.12	7.64 ± 2.10	7.92 ± 2.36	9.75 ± 2.45	9.77 ± 2.46	10.24 ± 2.41	0.001(27.36)*	0.69 (0.374)	0.97 (0.03)
JoLO	19.71 ± 6.87	17.68 ± 6.43	17.08 ± 6.34	20.41 ± 6.38	22.06 ± 5.78	20.29 ± 7.26	0.021(5.45)*	0.63 (0.47)	0.39 (0.96)

Data were represented as mean ± standard deviation; * indicates a significant difference between the two groups (*P* < 0.05). PD, Parkinson disease; MCI, mild cognitive impairment; HC, healthy control; M, male; F, female; UPDRS, Unified Parkinson’s Disease Rating Scale; H-Y, Hoehn-Yahr; LEED, levodopa equivalent daily dose; MoCA, Montreal Cognitive Assessment; TMT, Trail Making Test; Similarity, Semantic Similarity Test; HVLT-R, Hopkins Verbal Learning Test-Revised; VFT, Verbal Fluency Test; JoLO, Benton Judgment of Line Orientation test.

### Clinical Assessment

The severity and stage of PD were assessed using the Movement Disorder Society-Unified Parkinson’s Disease Rating Scale-III (MDS-UPDRS-III) and the H&Y scale, respectively. The levodopa equivalent daily dose (LEDD) in patients with PD-MCI was calculated as advised by Tomlinson et al. (2010). The global cognitive function of all subjects was scored using the MoCA.

Impairments in different cognitive domains were assessed using the following neuropsychological battery: (I) attention, Trail Making Test (TMT)-A, and TMT-B; (II) executive function, Semantic Similarity Test (Similarity); (III) episodic memory, Hopkins Verbal Learning Test-Revised (HVLT-R); (IV) language, Verbal Fluency Test (VFT); (V) visual–spatial function, Benton Judgment of Line Orientation test (JoLO).

### Data Acquisition

All MR experiments were performed using a 3.0-Tesla MR scanner (Discovery MR750w, GE Medical Systems, Milwaukee, WI, United States) with a standard 8-channel head coil. All participants wore headphones and were instructed to lie in a supine position.

Functional images were acquired using a gradient echo echo-planar imaging sequence with the following parameters: repetition time (TR), 2000 ms; echo time (TE), 30 ms; flip angle, 90°; slice thickness, 4 mm without gap; field of view (FOV), 240 mm × 240 mm; matrix size, 64 × 64; voxel size, 3.75 mm × 3.75 mm × 4.0 mm; and number of time points, 240. Each frame included 35 continuous slices that covered the whole volume of the brain. The slices were parallel to the line connecting the anterior commissure and posterior commissure. The fMRI scan took 5 min. As an anatomical reference, high-resolution T1-weighted images were acquired using a whole-brain three-dimensional brain volume imaging sequence with the following parameters: TR, 12 ms; TE, 5.1 ms; TI, 450 ms; flip angle, 15°; slice thickness, 1 mm without gaps; FOV, 240 mm × 240 mm; matrix size, 256 × 256; voxel size, 1 mm × 1 mm × 1 mm; number of slices, 172. All subjects were required to lie awake with their eyes closed and avoid thinking of anything during the MRI scan.

### Data Preprocessing and Analysis

The fMRI data were preprocessed using the Data Processing Assistant for Resting-State fMRI (DPARSF, version 4.3)^1^ (Chao-Gan and Yu-Feng, 2010), which is based on Statistical Parametric Mapping (SPM, version 12)^2^ and the Data Processing and Analysis of Brain Imaging toolbox (DPABI, version 3.0)^3^ (Yan et al., 2016) running in MATLAB R2016b (MathWorks Inc., Natick, MA, United States).

The preprocessing consisted of slice-timing correction, realignment, normalization, and smoothing. First, the first 10 time points from each subject were discarded to allow for patient adaptation and magnetic saturation effects. Slice-timing correction and head-motion correction were then performed. Subjects, whether patients or HC, were excluded if their head motion exceeded 2 mm in one or more linear directions or an angular rotation of 2°. Next, the individual T1-weighted images were co-registered to the mean realigned functional image generated during realignment and then segmented into gray matter (GM), white matter (WM), and cerebrospinal fluid (CSF) tissue maps using a unified segmentation algorithm. The images were then non-linearly normalized into the Montreal Neurological Institute (MNI) space using the diffeomorphic anatomical registration through exponentiated Lie algebra (DARTEL) approach. After normalization, functional volumes were resampled into a voxel size of 3 mm × 3 mm × 3 mm and spatially smoothed using a Gaussian filter with a full width at half-maximum (FWHM) of 6 mm. In addition, nuisance covariates (including the Friston-24 parameters, WM signal, CSF signal, and global mean signal) were regressed out following the preprocessing. The resulting fMRI data were then temporally bandpass filtered (0.01 Hz < *f* < 0.08 Hz) to reflect the actual spontaneous neural activity of the brain.

A seed-based correlation approach was performed. We used the bilateral caudate, defined by the automated anatomical labeling (AAL) atlas, as seed regions (Tzourio-Mazoyer et al., 2002) and resampled the data to 3 mm × 3 mm × 3 mm. The average time courses of individuals for each seed region were extracted at the voxel level. Correlation analysis was performed between the seed region and the entire brain. The correlation coefficients for the individual voxels were then converted to *z*-value maps by using the Fisher *Z* transformation.

### Genotyping

All participants’ genomic DNA was isolated from peripheral blood samples (2 ml) using QIAamp DNA Blood Kits (Qiagen, Hilden, Germany). Two single-nucleotide polymorphisms (SNPs), namely, rs7412 and rs429358, were genotyped to differentiate ε2, ε3, and ε4 allele (Williams-Gray et al., 2009). A polymerase chain reaction-based restriction fragment length polymorphism (PCR-RFLP) assay was applied to detect the alleles of rs7412 (forward primer: [FAM] GGCGCGGACATGGA GGAC, reverse primer: GCCCCGGCCTGGTACACT) and rs429358 [forward primer: (FAM) AGGGCGCTGATGGAC GAGAC, reverse primer: GCCCCGGCCTGGTACACT]. The procedures were exactly as described in a previously published protocol (Sun et al., 2017). According to a previous study (Chen et al., 2015) and the result of genotyping, the PD-MCI and HC groups were divided into ε2 (ε2 carriers), ε3 (ε3 homozygotes), and ε4 (ε4 carriers) subgroups, respectively.

## Statistical Analysis

The Mann–Whitney *U* test or Student’s *t* test (for continuous variables) and the χ^2^ test (for categorical variables) were utilized to investigate the differences in demographic data between groups. One-way analysis of variance was utilized to investigate the differences in demographic data (disease course, UPDRS, H&Y scale, LEED) between subgroups of PD-MCI patients. The χ^2^ test was performed to explore the distribution of genetic data. The above statistical analyses were performed using SPSS 19.0 software (version 19.0, SPSS Inc., Chicago, IL, United States). A *P* value < 0.05 was considered statistically significant.

Full factorial analysis was conducted to analyze the main effects and interaction effect of diagnosis and genotype using SPSS 19.0 (for demographic data and neuropsychological performance) and SPM 12 (for functional data). Specifically, the between-subject factors included diagnostic group (PD-MCI and HC) and genotype group (ε2, ε3, and ε4). The covariates for functional data analyses included gender, age, education, LEED, and modulated individual GM volumes from the preprocessing. The non-stationary cluster-level familywise error (FWE) method was used to correct for multiple comparisons, resulting in a voxel-wise height threshold of *P* < 0.001 (uncorrected) combined with a cluster-level extent threshold of *P* < 0.05 (corrected for multiple comparisons using the FWE rate).

For regions with any significant effect of functional data, Fisher’s *Z*-transformed FC strengths for each subject were extracted. *Post hoc* analysis for significant interaction effects was subsequently conducted using Bonferroni correction. The correlations between these *Z*-transformed FC strengths of regions and neuropsychological performance measures were investigated separately using Pearson correlation analysis in SPSS 19.0 software. The threshold for statistical significance was defined as *P* < 0.05. The covariates for the correlation analyses included gender, age, education, LEDD, and modulated individual GM volumes.

## Results

### Demographic and Cognitive Characteristics

There were no significant differences observed in age, gender, or years of education between groups or subgroups. The disease course and scores on the UPDRS, H-Y scale, and LEED were not significantly different among subgroups of PD-MCI (*P* = 0.56, *P* = 0.50, *P* = 0.98, *P* = 0.75). None of the ε24 heterozygotes was found among the subjects. The distribution of the ApoE genotype in the PD-MCI group was the same as in the HC group (*P* = 0.35). Significant main effects of diagnosis were observed for general cognition, executive function, episodic memory, language, and visual–spatial function. No significant main effect of genotype or interaction effect of genotype and diagnosis was found in any of the cognitive domains. Participant demographic and clinical data are shown in Table 1.

### Differences in Group-Level and Genotype-by-Disease Interaction of FC Analysis

**FIGURE 1:**
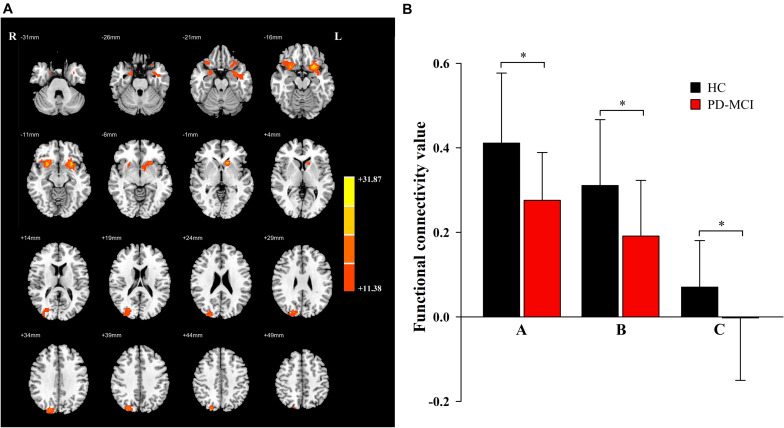
The main effects of diagnosis. Significant main effects of diagnosis **(A)** include bilateral inferior orbit frontal gyrus (OFG) and right superior occipital gyrus (SOG). The color bar represents the range of *F* values in the clusters **(B)**. The *Y*-axis represents FC values (mean ± standard deviation) extracted from regions with main effect of diagnostic. The *X*-axis represents regions with main effect of diagnostic group (right inferior OFG-A, left inferior OFG-B, right SOG-C). * indicates a significant difference between the two groups (*P* < 0.05). PD, Parkinson disease; MCI, mild cognitive impairment; HC, healthy control.

Significant main effects of diagnosis (Figure 1) were discovered between the bilateral caudate and the bilateral inferior orbitofrontal gyrus (OFG) and right superior occipital gyrus (SOG). Compared to the HC group, patients with PD-MCI showed significantly decreased FC between these regions (Figure 1). No significant main effect of genotype was revealed.

**FIGURE 2:**
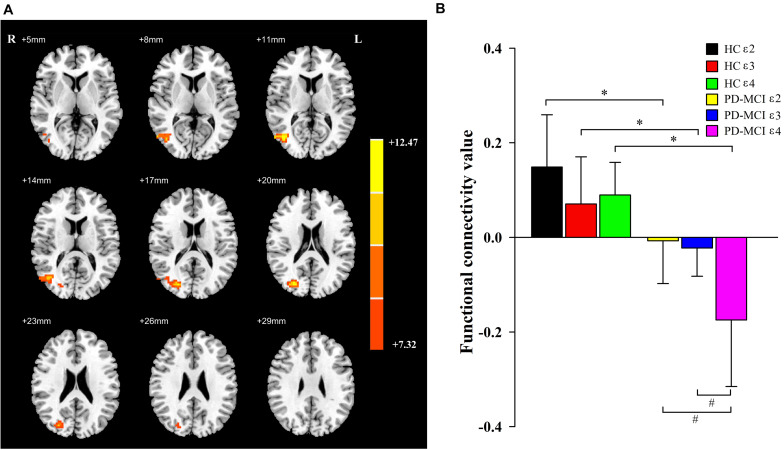
The main effects of interaction. Significant main effects of interaction **(A)** include right middle occipital gyrus and right middle temporal gyrus. The color bar represents the range of *F* values in the clusters **(B)**. The *Y*-axis represents FC values (mean ± standard deviation) extracted from regions with main effect of interaction. The *X*-axis represents regions with main effect of diagnostic group. * indicates a significant difference between the two subgroups of the same genotype carrier (*P* < 0.05). ^#^ indicates a significant difference between the two subgroups of the same group (*P* < 0.05). PD, Parkinson disease; MCI, mild cognitive impairment; HC, healthy control.

A significant interaction effect (Figure 2) was discovered for FC between the bilateral caudate and the right middle occipital gyrus (MOG) and right middle temporal gyrus (MTG). Compared to the HC group, patients with PD-MCI showed significantly decreased FC between these regions (Figure 2). Furthermore, the FC value of ε4 carriers was significantly lower than those of ε2 or ε3 carriers in the PD-MCI group (*P* = 0.028, *P* = 0.010). There were no significant differences observed between ε2 carriers and ε3 carriers in either the PD-MCI group or the HC group (*P* > 0.05). The main effect of diagnosis and the interaction effect of genotype and diagnosis on FC are listed in Table 2.

**TABLE 2:** Descriptions of brain regions for the main effect of diagnosis and the interaction effect of genotype and diagnosis on caudate FC.

Brain regions (AAL)	Peak MNI coordinates (mm)			Peak *F* value	Cluster size (mm^3^)
	*X*	*Y*	*Z*		
**Main effect of disease**					
Left inferior orbit frontal gyrus	−12	18	0	24.98	348
Right inferior orbit frontal gyrus	27	15	−12	25.85	244
Right superior occipital gyrus	24	−84	42	22.85	211
**Disease × genotype interaction**					
Right middle occipital gyrus/Right middle temporal gyrus	42	−72	12	12.47	158

*These findings correspond to a voxel-wise height threshold of *P* < 0.001 (uncorrected) combined with a cluster-level extent threshold of *P* < 0.05 (corrected for multiple comparisons using the FWE rate). AAL, automated anatomical labeling; MNI, Montreal Neurological Institute.*

### Correlation Analysis

**FIGURE 3:**
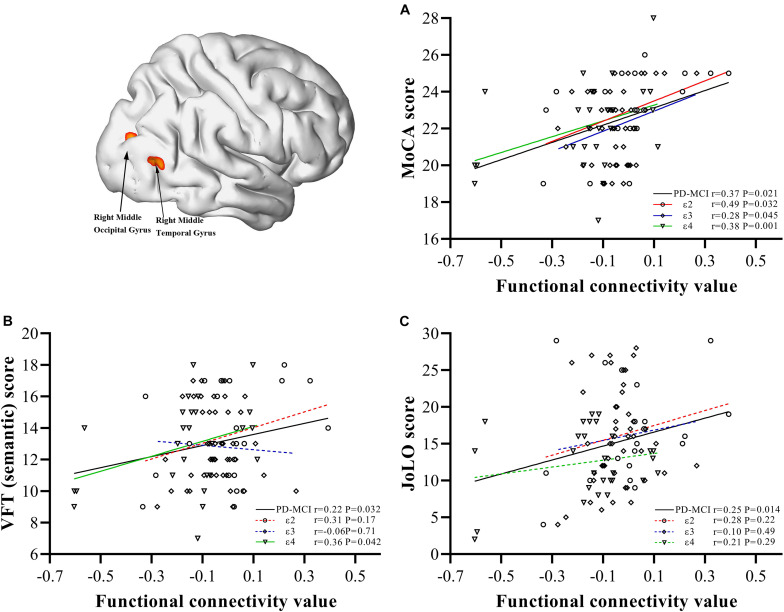
Correlations between abnormal bilateral caudate functional connectivity values extracted from regions with main effect of interaction and neuropsychological characteristics. Scatter plots demonstrate the FC value of regions with main effect of interaction (right middle occipital gyrus/right middle temporal gyrus) and scores of MoCA **(A)**, semantic fluency performance **(B)**, JoLO **(C)** in the PD-MCI group. The solid line represents significant correlation, whereas the dotted line represents non-significant correlation. PD, Parkinson disease; MCI, mild cognitive impairment; HC, healthy control; MoCA, Montreal Cognitive Assessment; VFT, Verbal Fluency Test; JoLO, Benton Judgment of Line Orientation test.

In the PD-MCI group (Figure 3), FC values between the bilateral caudate and right MOG and right MTG were significantly positively correlated with MoCA (*r* = 0.37, *P* = 0.021), semantic fluency performance (*r* = 0.22, *P* = 0.032), and JoLO (*r* = 0.25, *P* = 0.014) scores. For the subgroups, we found a significantly positive correlation between the FC and MoCA scores of each subgroup (ε2, *r* = 0.49, *P* = 0.032; ε3, *r* = 0.28, *P* = 0.045; ε4 *r* = 0.38, *P* = 0.001), but only the FC of ε4 carriers was found to be significantly positively correlated with semantic fluency scores (*r* = 0.36, *P* = 0.042). No FC variable was significantly correlated with JoLO scores in any of the three subgroups (*P* > 0.05).

## Discussion

In this study, by using FC and genotyping, we obtained multiple significant findings. (i) Significantly decreased FC between the caudate and the bilateral inferior OFG and bilateral MOG was found in patients with PD-MCI compared to HC, along with poor performance in general cognition, executive function, episodic memory, language, and visual–spatial function. (ii) Significantly decreased FC between the caudate and right MOG and right MTG was found as an interaction effect. (iii) For patients with PD-MCI, the FC values of ε4 carriers were much lower than those of ε2 or ε3 carriers, and FC was positively correlated with the impairment of global and language function. These findings provide promising evidence that the ε4 allele of the ApoE gene is associated with functional dysconnectivity between the caudate and other brain regions in an interactive manner.

Changes in the striatum, the region composed of the caudate and putamen, are related to heterogeneous symptoms in PD, as supported by a number of studies (Wu et al., 2010; Ekman et al., 2012; Luo et al., 2014; Manza et al., 2016). In particular, the caudate nucleus is regarded as a subcortical hub for cognitive function (Manza et al., 2016). In our study, a main effect of diagnosis was observed, with decreased FC in the bilateral inferior OFG (part of the prefrontal cortex) and poor performance in executive function. These findings are consistent with previous studies proposing that the caudate nucleus is organized into cortico-striato-thalamo-cortical loops connecting to the prefrontal cortex, which mediate some forms of executive function (Cools, 2008; Zhang et al., 2012; Manza et al., 2015).

Multiple cognitive domains are frequently affected in PD. Recent evidence indicates that cholinergic dysfunction within the posterior cortex is a predictor of progression toward prodromal PDD (Kehagia et al., 2010, 2013; Ekman et al., 2014; Wolters et al., 2019), whereas dopaminergic dysfunction in the frontostriatal circuits is not (Kehagia et al., 2013; Winder-Rhodes et al., 2013). From a longitudinal PET study, (Pappata et al., 2011) found significant hypometabolism in the associated occipital cortex and neostriatum in PD-MCI patients relative to healthy subjects. We identified the bilateral MOG (part of the occipital lobe) as another diagnostic brain region of vital importance for the functions examined in our study, as well as for visual–spatial processing, as reported in previous studies (Yang et al., 2013; Fang et al., 2017). Moreover, our results showed that the MTG was a significant region involved in an interactive effect on language impairment in PD-MCI. Our study was also consistent with the findings from other neuroimaging studies (Hou et al., 2016; Tang et al., 2016), which have focused on the involvement of the temporal lobe in PD-MCI. Therefore, our findings support the hypothesis that early involvement of posterior cortical regions is an early marker of dementia.

Apolipoprotein E is the most abundant apolipoprotein in the central nervous system, and its secretion by astrocytes is responsible for neuronal cholesterol homeostasis, neuronal growth, membrane plasticity, and synapse development (Emamzadeh, 2017). Recent studies have revealed that ApoE is associated with cognitive decline but not the development of PD (Peplonska et al., 2013; Multhammer et al., 2014). Gallardo et al. (2008) found that ApoE ameliorates amyloid-β (Aβ) accumulation and neurodegeneration in a transgenic mouse model of PD and indicated that the interaction of ApoE with distinct neural substrates produced some of the alterations observed in PDD. Nombela et al. (2014) found reduced brain activity within the temporal lobe and impaired visual–spatial and memory performance in carriers of ε4. However, the association between brain activity and genetic effects was not further studied. In our study, a significant FC alteration within the posterior cortex was identified by fMRI as an interaction effect, providing evidence that the impairment of cognition-related posterior cortex functions is not simply an effect of PD but an interaction effect between ApoE genotype and PD. Additionally, although the diagnosis of PD-MCI relies on neuropsychological examinations, we were able to observe significant interactions using FC analysis rather than neuropsychological scales while in the OFF state. Thus, we hypothesized that rs-fMRI could be a robust surrogate to predict the cognitive decline that marks PD-MCI in the OFF state. However, the validation of rs-fMRI requires further investigation in our future work, when taking the effect of dopaminergic therapy on FC into account.

Apolipoprotein E isoforms are associated with Aβ plaque deposition (Irwin et al., 2013; Paul et al., 2016) and α-synuclein aggregation (Emamzadeh et al., 2016; Emamzadeh, 2017) in PD, with ε4 considered to carry a higher risk than either of the others (ε2 and ε3). Paul et al. (2016) and Tropea et al. (2018) examined associations between genes and PD susceptibility and found that ε4 carriers exhibit faster decline than non-carriers in many neuropsychological test scores. In our study, by taking an fMRI-based approach that is sensitive to functional alterations, we observed that the FC values of ε4 carriers with PD-MCI were much lower than those of ε2 or ε3 carriers and were positively correlated with performance on the VFT, which corroborates the findings of Mata et al. (2014); Wisdom et al. (2011) conducted a meta-analysis of 77 studies, including a total of 40,942 cognitively healthy adults, and found that ε4 carriers performed significantly worse than non-carriers in all tested domains except verbal ability, visual–spatial skill, and attention. Although the assessment of language and visual–spatial function was too brief to be investigated precisely in our study, we still found that ApoE genotype was associated with impairment of semantic fluency performance and visual–spatial function, which differed from the background effect of ApoE in the general population. Compared to MCI in AD, PD-MCI frequently involves an increased severity of executive and visual–spatial deficits and a decreased severity of memory impairment (Aarsland, 2016; Jellinger and Korczyn, 2018). Overall, our fMRI study provided initial evidence that the ε4 genotype is associated with increased risk, and we hypothesized that the posterior cortex was involved in the interaction, differing from the regions involved in AD. Further studies on the precisely interactive effects of ApoE genotype are required.

Although the FC values between the bilateral caudate and right MOG and right MTG decreased dramatically, the subgroups were not entirely consistent with each other. The present study also observed marked correlations between FC values and some cognitive domains (global, language, and visual–spatial function) in the PD-MCI group. However, the subgroups based on the ApoE subtypes were not consistent. Thus, our results could indicate that genetic factors affect the patterns of FC changes and contribute to the cognitive heterogeneity of PD-MCI cases. Additionally, Biundo et al. (2011) characterized the relationship between ApoE status and residual semantic abilities in amnestic MCI with a category fluency task, and the results indicated that patients were highly risky for impaired semantic fluency performance in comparison to non-ε4 carrier of MCI subjects. Together with our results revealed by rs-fMRI, it could be deduced that semantic fluency, together with posterior cortical involvement and status of ε4, may underline not just a cognitive decline in PD but represents an “interplay model” that increases risk for AD co-pathology in PD.

Several limitations of this study should be recognized. First, although we grouped all subjects into three subgroups according to their ApoE isoforms, the sample size in this study was insufficient and may introduce a statistical bias in our results. Thus, additional cases are required to assess the interaction effect in future studies. Second, as a preliminary study, each cognitive profile was assessed with only one scale, which hindered the precise diagnosis of PD-MCI, analysis of the domains, and the correlation to altered FC. More detailed neuropsychological scales corresponding to different cognitive domains would be better for understanding the underlying mechanisms in cognitive dysfunction. Third, this was a cross-sectional study comparing PD-MCI patients and HC. Hence, our results should be confirmed by a future longitudinal study over the progression of PD, including PD with normal cognition, and PDD. Fourth, only ApoE was included in our study. Brain connectivity might be influenced by other genes associated with CI in PD, such as SNCA, catechol-*O*-methyltransferase, and MAPT, although there remains controversy regarding these genes. Fifth, in order to identify the interaction effects through both the clinical neuropsychological scales and the FC approach, we used a strict significance threshold that may have caused some false negatives in individual comparisons. Finally, although no significant difference in LEED was found among the subgroups of PD-MCI, the effect of antiparkinsonian medication should be considered in future studies.

In conclusion, this preliminary study verified that altered FC between the bilateral caudate and posterior cortical regions was interactively influenced by ApoE genotype and PD-MCI status. Importantly, the ε4 subtype is a risk factor for rapid global and verbal cognitive decline in PD-MCI. Our findings provide the first fMRI evidence relating ApoE polymorphism to cognitive decline in PD, and they highlight the credibility of fMRI as a source of neuroimaging markers to explore the genetic basis of pathophysiology. Further longitudinal studies will be necessary to confirm the mechanisms through which ApoE ε4 contributes to the risk of PD-MCI.

## Data Availability Statement

The original contributions presented in the study are publicly available. This data can be found here: https://figshare.com/s/4bd103a94e36f97ad23d.

## Ethics Statement

The studies involving human participants were reviewed and approved by The local institutional review board of Clinical Medical College, Yangzhou University (2015KY-081). The patients/participants provided their written informed consent to participate in this study.

## Author Contributions

SS, HZ, and JW contributed to the data collection. SS, Y-CC, and HZ contributed to PI. SS, WD, LQ, XY, and JW contributed to the protocol and statistics. SS, WD, LQ, and XY contributed to the analysis. SS and Y-CC contributed to manuscript writing. All authors contributed to the article and approved the submitted version.

## Conflict of Interest

WD and LQ are employed by the company MR Research China, GE Healthcare. The remaining authors declare that the research was conducted in the absence of any commercial or financial relationships that could be construed as a potential conflict of interest.

## Footnotes

1 http://rfmri.org/DPARSF

2 https://www.fil.ion.ucl.ac.uk/spm

3 http://rfmri.org/DPABI
